# The Practitioner's Relaxations and Hobbies

**Published:** 1911-12-23

**Authors:** 


### THE PRACTITIONER'S RELAXATIONS AND HOBBIES.

### A Surgeon in Ethiopia.

ONLY a year or two ago Sir Frederick Treves spent some of the leisure, which has been his since he retired from active practice, in a visit to British East Africa and Uganda; and he published in book form his impressions of the fascinating countries in which he toured. Since then two more London surgeons, Mr. J. Bland Sutton and Dr. Comyns Berkeley, have imitated Sir Frederick, though in their case the necessary time had to be snatched during the intervals of busy hospital and other professional labours. The former of these well-known Middlesex and Chelsea Hospital colleagues has now still further followed the example of Sir Frederick by issuing in book form the account, not so much of his own individual wanderings and impressions, as of the natural history and ethnology of the wild creatures of the Protectorate.*

As a general rule, the wandering tourist who rushes into print on some foreign people or foreign country after a brief sojourn with them commits himself to many ill-advised or erroneous opinions. One cannot acquit Mr. Sutton of the charge of being a wandering tourist, but fortunately his whole training and character have constituted him a careful and accurate observer. Bearing in mind the dexterity wherewith he made interesting the dry subject of "ligaments" to the medical students of the nineties, it would be very astonishing if he failed to transmit to his readers the inherent fascination of the continent of which it is truly written *semper aliquid novi*. In this particular case, it happens, the author has very little that is new, or even novel, to relate; but that is chiefly because he has trusted almost entirely to competent authorities for his facts.

The consequence is that the volume is better suited for the ordinary reader than for the experienced (East African) traveller already familiar with the standard works which Mr. Sutton has consulted. The author's experience as a writer of text-books for students has doubtless stood him in good stead in this respect; and his industry in collating every available special monograph on the various matters with which his chapters deal, has resulted in an accumulation of facts perhaps greater than in any other work of the same size dealing with this part of Africa.

Let it not be supposed that Mr. Sutton fails to impress his own individuality on the pages of his book; so vigorous and distinct a personality runs little fear of that risk, though "Suttonisms," if he will pardon the expression, are much fewer than might have been expected. There is a full and conscientious list of the ten ornaments, charms, etc., worn by an otherwise perfectly nude Kavirondo tribesman, and an explanation of the object with which each was adopted. This list is taken from a monograph by Mr. Hobley, one of the very first-rank authorities on the subject. On turning the page there follows a list of ten more ornaments, charms, etc., worn by a London fashionable lady who happened to call on Mr. Sutton just as he wrote this particular chapter. Without in the least straining after effect, he is easily able to compare the items of the two lists and to demonstrate the amazing resemblance between them.

It has been pointed out that Mr. Sutton's facts are mainly obtained from first-class authors—the standard text-books of the subject, in fact. On rare occasions he deserts these trustworthy guides and sometimes makes a slip. Thus he describes the front horn of a rhinoceros as rarely exceeding one foot in length, a mistake the more surprising in that the author's long connection with the London Zoological Gardens renders him as a rule exceptionally well informed on points of natural history.

The public interest in ethnology is steadily growing, and the attention devoted to the tribes of East Africa in many recent books on that region is an excellent sign of the times. Without carrying his own enthusiasm to the length of boring the reader, Mr. Sutton manages to convey a lot of information on this subject. In describing the peculiarly disgusting way in which the Kavirondo pursue the "sour milk treatment" he might perhaps mention that the Masai and some other allied tribes do it also. The custom of adorning the top of the hut with an upturned crock or pot as a charm to prevent the owner's babies from squinting is also not confined to the Kavirondo, for the Elgeyo people do exactly the same thing.

In books of this scope the illustrations are always an important item. Mr. Sutton has reverted to the old-fashioned woodcut, but he has had the drawings done from photographs, for the most part. To be successful, such engravings require to be extremely well done, and then they often surpass even the best photographs. In the present case candour compels one to admit that the artist has not reached the highest level of excellence, and that several of his drawings do not give a correct representation of the animals and men they depict. Amongst the tribesmen, noticeably, there is no attempt to express the very marked differentiation of features which exists as between one race and another; and the animals, too, are often badly and incorrectly drawn. This is a pity, because there are artists who have personal experience of tropical Africa and its denizens, and combine excellence in technique with an expert appreciation of their subjects. It is only necessary to mention the name of Mr. Caldwell in this connection to emphasise this point. Anyone who compares the drawing of a waterbuck on p. 296 of this book with that of the same animal in Sir Percy FitzPatrick's "Jock of the Bushveld" will realise this point; and to those who know this lovely animal in his haunts the difference is greater still.

---

* *Man and Beast in Eastern Ethiopia*, by J. Bland Sutton, F.R.C.S. London: Macmillan. 1911. Price 12s. net.

